# Evaluation of type 1 diabetes’ partial clinical remission after three years of heterologous adipose tissue derived stromal/stem cells transplantation associated with vitamin D supplementation

**DOI:** 10.1186/s13098-024-01302-2

**Published:** 2024-05-24

**Authors:** Isabella Sued Leão, Joana Rodrigues Dantas, Débora Baptista Araújo, Maria Eduarda Nascimento Ramos, Karina Ribeiro Silva, Leandra S. Batista, Maria de Fátima Carvalho Pereira, Ronir Raggio Luiz, César Cláudio da Silva, Angelo Maiolino, Carmen Lúcia Kuniyoshi Rebelatto, Débora Regina Daga, Alexandra Cristina Senegaglia, Paulo Roberto Slud Brofman, José Egídio Paulo de Oliveira, Lenita Zajdenverg, Melanie Rodacki

**Affiliations:** 1 Department of Internal Medicine, Nutrology Section, Universidade Federal do Rio de Janeiro (UFRJ), Av Professor Rodolpho Paulo Rocco 255, 22440035 Rio de Janeiro, RJ Brazil; 2 Laboratory of Tissue Bioengineering, Instituto Nacional de Metrologia Qualidade e Tecnologia Campus de Xerem, Duque de Caxias, Brazil; 3 Histology and Embryology Departament, Universidade Estadual do Rio de Janeiro, Rio de Janeiro, Brazil; 4 Center for Biological Research (Numpex-Bio), Universidade Federal do Rio de Janeiro, Rio de Janeiro, Brazil; 5 Institute of Public Health Studies, Universidade Federal do Rio de Janeiro, Rio de Janeiro, Brazil; 6 Plastic Surgery, Universidade Federal do Rio de Janeiro, Rio de Janeiro, Brazil; 7 Hematology Department, Universidade Federal do Rio de Janeiro, Rio de Janeiro, Brazil; 8 Core Cell Technology, Pontifical Catholic University of Parana, Curitiba, Brazil

**Keywords:** Diabetes mellitus type 1, Stem cells, Vitamin D, Partial remission, Honeymoon phase

## Abstract

**Background:**

Mesenchymal stem cell infusion and vitamin D supplementation may have immunomodulatory actions that could prolong the preservation of residual insulin secretion in patients with type 1 diabetes (T1D). Intervention with these agents after onset of T1D could favor the development of a remission phase, with potential clinical impact. We aimed to compare the presence of clinical remission (CR), glycemic control and daily insulin requirement at 6, 12, 18, 24 and 36 months after the diagnosis of T1D using IDAA1c in patients who received therapy with adipose tissue-derived mesenchymal stem cell (ASC) infusion and vitamin D supplementation and a control group. Methods: This retrospective cohort study analyzed data from the medical records of patients with T1D diagnosed between 15 and 40 years. Partial CR was defined as an IDAA1c index < 9. Patients in the intervention group received an infusion of adipose tissued-derived mesenchymal stem cells (ASCs) within 3 months after diagnosis and supplementation with 2000 IU of cholecalciferol for 1 year, started on the day following the infusion. Partial CR was also determined using the ISPAD criteria, to assess its agreement with IDAA1c. Results: A total of 28 patients were evaluated: 7 in the intervention group (group 1) and 21 in the control group (group 2). All patients in group 1 evolved with partial CR while only 46.7% of patients in group 2 had this outcome. Group 1 had a higher frequency of CR when evaluated with IDAA1c and ISPAD criteria. The mean duration of CR varied between the two criteria. Although HbA1c was similar between groups during follow-up, group 1 had a lower total daily insulin requirement (*p* < 0.005) at all time points. At 36 months, group 1 used 49% of the total daily insulin dose used by group 2 with similar glycemic control. Conclusion: The intervention with infusion of ASC + vitamin D supplementation was associated with partial CR at 6 months. Although there were no differences in CR established by the IDAA1c and ISPAD criteria after three years of follow-up, patients who underwent intervention had nearly the half insulin requirement of controls with conventional treatment, with similar glycemic control.

**Trial Registration:**

37001514.0.0000.5257.

## Background

Type 1 diabetes mellitus (T1D) is a chronic disease characterized by immune-mediated destruction of pancreatic beta cells, which leads to a progressive decrease in insulin secretion [1]. When approximately 65–80% of pancreatic beta cells are destroyed, patients usually present the classic symptoms of the disease, such as polyuria, polydipsia and weight loss, and require exogenous insulin replacement [2].

After clinical diagnosis and initiation of insulin therapy, some patients may experience a transient recovery of pancreatic β-cell function, which may be partial or complete. This period is known as clinical remission (CR) or the “honeymoon” phase [3]. The clinical remission phase is characterized by satisfactory glycemic control with low or any insulin requirement. A longer duration of this phase is associated with better subsequent glycemic control during follow-up [4], as well as a lower risk of chronic complications and severe hypoglycemia [5].

Numerous interventions are currently being tested to achieve the preservation of residual insulin secretion, T1D cure or the development of CR, such as immunosuppressive agents, immunomodulators and autoantigens for immune induction, with variable results [6]. Stromal stem cells (SSCs) are particularly interesting candidates for the treatment of T1D. SSCs show immunomodulatory effects in other immune-based diseases and do not have stimulatory molecules for self-reactive T cells. Thus, they can be used in allogenic transplants without the need for immunosuppression and with few reported complications [7, 8]. Adipose tissue may represent an abundant source of SSCs. The potential application of adipose tissue-derived stromal/stem cells (ASCs) as an alternative source to bone marrow SSCs is of great interest, since the latter require immunosuppression, with a greater risk of severe complications [9]. Vitamin D supplementation has also been extensively studied in the last decade due to its significant role in the immune system, both in the adaptive and innate response, and may also influence pancreatic β cells, which have receptors for vitamin D [10, 11].

Due to the complexity of the pathophysiology of T1D, the combination of more than one intervention for the tertiary prevention of the disease has been studied. Araújo et al. previously demonstrated the short-term safety of ASC in combination with vitamin D for patients with newly diagnosed T1D as well as potential favorable results [12]. However, a longer follow-up and a larger control group are needed to evaluate the impact of this treatment in patients with recent-onset T1D. The aim of this study was to compare the period of CR during the first three years of T1D between patients who underwent intervention with ASC associated with vitamin D and insulin therapy versus those who followed standard insulin treatment.

## Methods

### Patient selection and study design

This retrospective cohort included patients from the T1D outpatient clinic from a University Hospital who were diagnosed with T1D between 16 and 40 years of age. Medical charts of patients who received a single stem cell infusion + daily vitamin D supplementation for 12 months as well as a control group were reviewed. Clinical and laboratory variables were retrieved to determine Insulin-Dose Adjusted A1c (IDAA1c) index, calculated as A1c (%) + 4x insulin dose (UI/kg) [13], the presence or absence of partial CR and its duration and glycated hemoglobin (HbA1c) at T1D onset and during follow-up (6, 12, 18, 24 and 36 months after diagnosis).

The inclusion criteria comprised patients with T1D follow-up in Federal University of Rio de Janeiro (UFRJ) hospitals and a diagnosis of T1D with ages between 16 and 40 years-old. The American Diabetes Association (ADA) criteria were used to define T1D [14]. Patients with insufficient data or diabetic ketoacidosis (DKA) at diagnosis were excluded from the study, as well as those with thyroid dysfunction, adrenal failure, chronic kidney disease or those who used corticosteroids. The control group was matched for gender and birth decade. Both groups received intensive basal bolus insulin treatment, according to ADA recommendation [14] and had insulin adjustments performed by the same care team according to glucose monitoring in each visit, which occurred every three to four months. All patients received the same diabetes education, nutritional recommendations, and help with management from healthcare providers.

### Partial clinical remission definition

Partial CR was determined by calculating the insulin-dose adjusted A1c (IDAA1c) index, performed 6 months after diagnosis. Values ​​below 9 are consistent with partial remission. Partial CR was also determined using the International Society of Pediatric and Adolescent Diabetes (ISPAD) criterion (HbA1C < 7.0% and total daily dose of insulin per kilogram of body weight < 0.5 IU/kg/day), to assess its agreement with the IDAA1c.

### Adipose tissue stromal/stem cell (ASC) infusion

Samples of adipose tissue cells were obtained through liposuction from three healthy women. The donors had negative serological tests for syphilis, Chagas disease, hepatitis B and C, HIV and HTLV. Those with IgG + for cytomegalovirus had negative polymerase chain reaction in blood and ASC samples. The samples were processed in the Core Cell Technology of the Pontifical Catholic University of Paraná as previously described [12]. In summary, 100 ml of adipose tissue was washed in sterile phosphate-buffered saline (PBS) (Gibco Invitrogen). A one-step digestion by 1 mg/ml collagenase type I (Invitrogen) was performed for 30 min at 37 °C during permanent shaking, followed by a filtration step through a 100 μm mesh filter (BD FALCON, BD Biosciences Discovery Labware). The cell suspension was centrifuged at 800 g for 10 min, and erythrocytes were removed through a lysis buffer with pH 7.3. The remaining cells were washed at 400 g for 10 min and then cultured at a density of 1 × 105 cells/cm2 in T75 culture flasks and DMEM-F12 (Gibco Invitrogen) supplemented with 10% of fetal calf serum, penicillin (100 units/ml), and streptomycin (100 µg/ml). The culture medium was replaced three days after seeding, and twice a week after that. ASCs were subcultured after reaching 80% confluence, with 0.5% trypsin/EDTA (Invitrogen) solution. Cells were related at a density of 4 × 103 cells/cm2 for expansion. On the day of infusion, the ASC monolayer were dissociated as described above, and 1 x Kg x 106 cells of the recipient patient were resuspended in 5 ml of saline solution with 50% albumin and 5% ACD (anticoagulant citrate dextrose solution). The cell suspension was sent to the hospital in a cooler with recycled ice. Patients that received ASCs were admitted to hospital on the day of the infusion and discharged 24 h after it. A single dose of ASCs was infused in a peripheral upper arm vein for 15–20 min. These patients started to use 2000 IU of oral cholecalciferol in the day after the infusion of ASCs.

### Laboratory analysis

To calculate the IDAA1c and evaluate partial CR using the ISPAD criteria, the HbA1c values at 6, 12, 18, 24 and 36 months after the diagnosis of T1D were retrieved. HbA1c was measured using the HPLC (*high performance liquid chromatography by boronate affinity*) method.

### Statistical analysis

Data are expressed as the mean ± standard deviation. Descriptive statistics were used to analyze patients’ characteristics. Comparison of categorical variables was performed using the Chi-square test. The Mann-Whitney test was applied to compare continuous variables between groups. Results with *p* ≤ 0.05 were considered statistically significant. SPSS software, version 21.0 was used for statistical analysis.

### Ethics statement

This study was approved by the Clementino Fraga Filho University Hospital (HUCFF)– Federal University of Rio de Janeiro (UFRJ)– Research Ethics Committee, protocol number 37001514.0.0000.5257. All patients signed the Informed Consent Form. In the case of minors, their legal guardians signed the consent form.

## Results

### Clinical characteristics of the study group

**Fig. 1:**
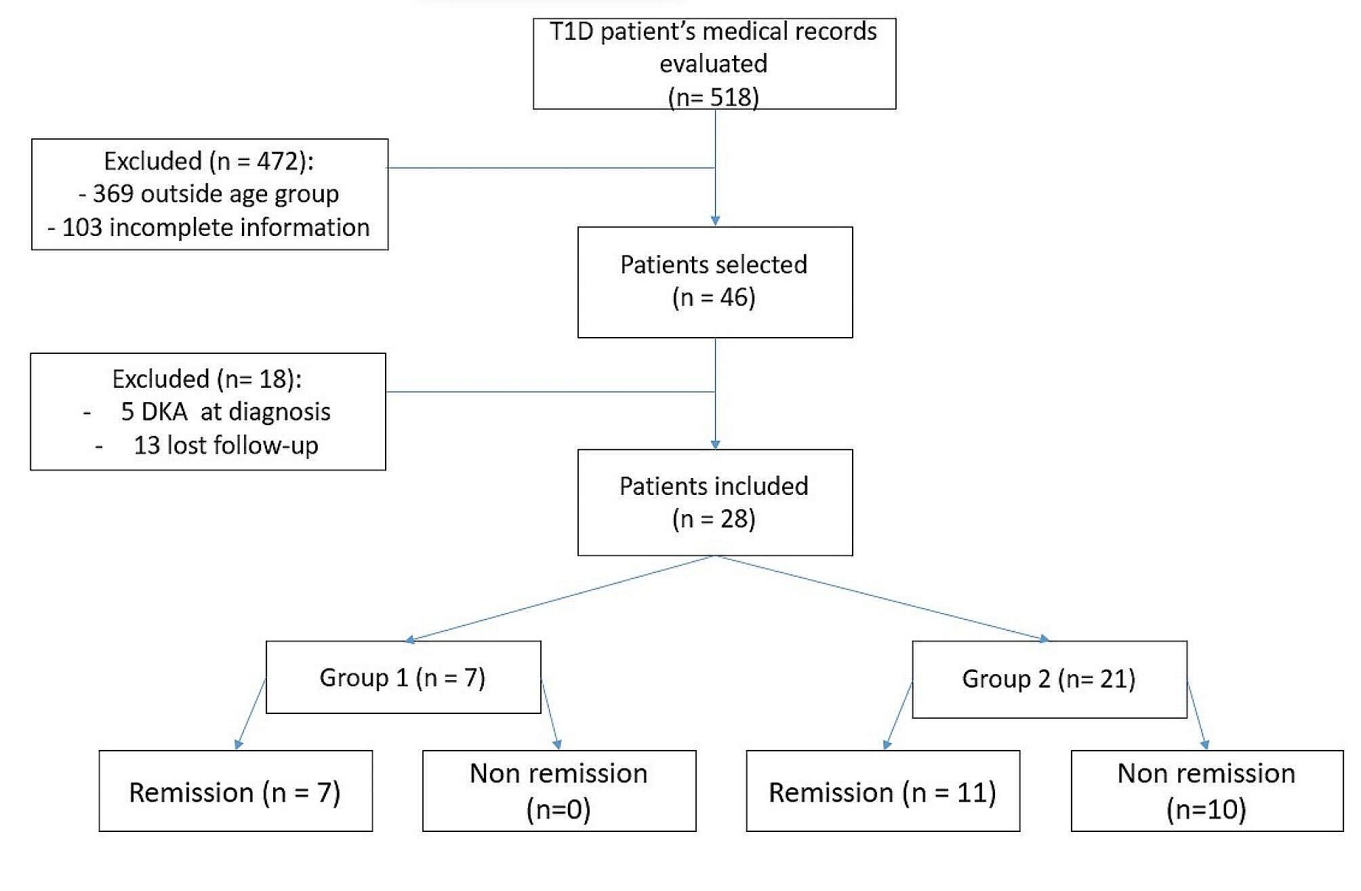
Flowchart: Patients recruited for the study, excluded and those who completed 36 months of follow-up. T1D: type 1 diabetes; DKA: diabetes ketoacidosis; Group 1: intervention group (adipose-stem cells infusion + vitamin D); Group 2: control group

A total of 518 medical records from the HUCFF T1D outpatient clinic were analyzed. Twenty-eight patients were selected, seven were allocated to the group that received the intervention (group 1) and twenty-one to the control group (group 2), as shown in Fig. 1. The main reasons for not including patients in the study were the age of T1D diagnosis lower than 16 years old and the lack of data availability of all periods studied in some patients diagnosed at an older age. One patient who received the intervention was excluded due to loss of follow-up.

All included subjects completed the 36-month follow-up. The clinical characteristics of each group are shown in Table 1.

**Table 1:** Clinical characteristics of the study groups

	Group 1	Group 2	*p* value
Age at T1D onset	27.28 (± 6.67)	21 (± 5.32)	**0.036**
Sex (female %)	57.14%	57.14%	1.0
BMI at T1D onset	22.68 (± 1.42) kg/cm2	19.35 (± 2.98) kg/cm2	**0.005**
HbA1c at baseline	9.7% (± 2.65)	10.23% (± 3.23)	0.4

T1D: Type 1 diabetes; BMI: body mass index; HbA1c: glycated hemoglobin; Group 1: intervention group; Group 2: control group

Age, BMI and HbA1c are expressed as the mean with standard deviation

Statistical analysis: Chi-square test for categorical variables and Mann-Whitney test for continuous variables

### ASC infusion and adverse events

ASC infusion was performed as previously described [15] and was associated with mild adverse events. All patients had transient headache and mild local infusion reactions. Tachycardia (*n* = 4) and abdominal cramps (*n* = 1) were also observed. Four patients developed local thrombophlebitis within the first week and two had transient mild eye floaters during infusion, with no subsequent visual abnormalities. One patient developed central retinal vein occlusion three months after the infusion, with complete resolution six months after the infusion. One patient had a recurrence of a benign ovarian teratoma with complete surgical removal, which was previously described [16]. No long-term adverse events were observed.

### Insulin daily dose and glycemic control

In group 1, the mean insulin dose remained stable from baseline until 12 months, with a mild increase in total insulin daily dose from 18 months until the end of follow-up. In group 2, the mean insulin dose at baseline increased progressively during follow-up. The total daily dose of insulin/kg was significantly lower in the intervention group (group 1) than in the control group (group 2), with no differences in glycemic control (Table 2). At 36 months, the intervention group used a 49% lower total daily insulin dose per kg than the control group.

**Table 2:** Comparison of glycemic control, total daily insulin dose, and partial CR according to IDAA1c and IDAA1c values between groups 1 and 2

	Group 1	Group 2	*p* value
**HbA1c** (%)			
6 months 12 months 18 months 24 months 36 months	6.58 (± 1.15) 6.91 (± 0.75) 7.24 (± 0.99) 7.44 (± 1.15) 7.7 (± 1.67)	6.37 (± 1.34) 7.5 (± 2.14) 8.04 (± 2.36) 8.20 (± 2.73) 7.75 (± 2.61)	0.917 0.917 0.466 0.678 0.725
**Total insulin dose/kg**			
6 months 12 months 18 months 24 months 36 months	0.26 (± 0.17) 0.30 (± 0.14) 0.40 (± 0.15) 0.49 (± 0.24) 0.45 (± 0.10)	0.58 (± 0.32) 0.67 (± 0.34) 0.76 (± 0.42) 0.84 (± 0.44) 0.91 (± 0.47)	**0.006** **0.001** **0.008** **0.008** **0.027**
**Partial CR (n and %)**			
6 months 12 months 18 months 24 months 36 months	7 (100%) 6 (85.7%) 4 (57.1%) 4 (57.1%) 3 (42.9%)	10 (47.6%) 10 (47.6%) 6 (28.6%) 7 (33.3%) 7 (33.3%)	**0.014** 0.078 0.172 0.264 0.373
**IDAA1c**			
6 months 12 months 18 months 24 months 36 months	7.54 (± 0.65) 8.15 (± 1.14) 8.77 (± 1.02) 9.40 (± 1.69) 9.50 (± 1.63)	9.14 (± 2.50) 10.29 (± 3.12) 11.39 (± 3.37) 11.58 (± 3.74) 10.95 (± 2.86)	0.101 0.113 **0.048** 0.113 0.219

HbA1c: glycated hemoglobin. CR: Clinical remission

All continuous variables are expressed as the mean with standard deviation

Statistical analysis: Mann-Whitney test; significance value: p < 0,05(bold)

### Partial clinical remission

**Fig. 2:**
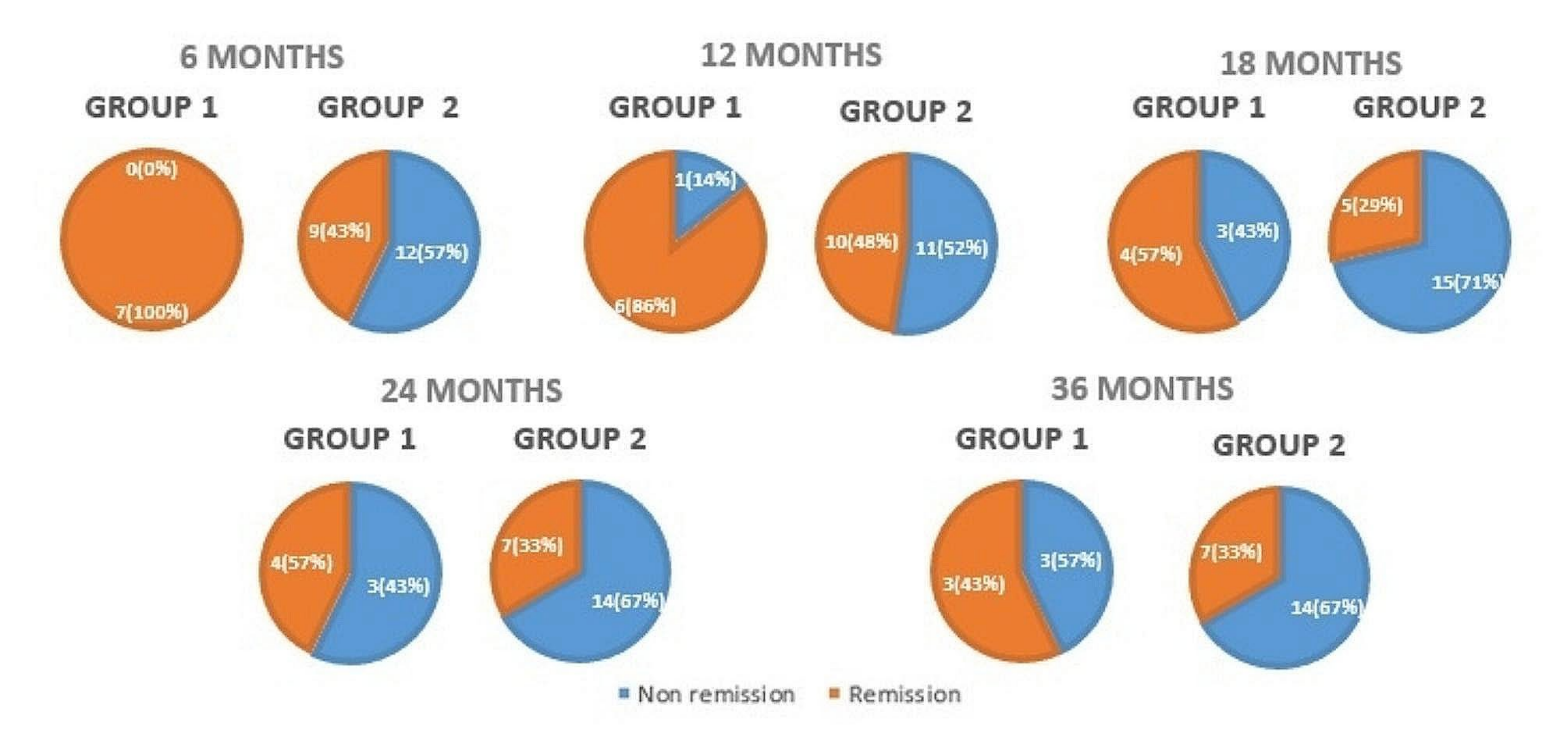
Partial clinical remission in group 1 and group 2 during follow-up– n (%). Group 1: intervention group (adipose-stem cells infusion + vitamin D); Group 2: control group. The figure shows the absolute number of patients and the percentage of patients in each group

According to the IDAA1c criteria, 100% of patients in group 1 evolved with partial CR while 47.6% of patients in group 2 had this outcome. At the first 6 months after diagnosis, group 1 presented with significantly more partial CR than group 2 (*p* = 0.014), without differences at 12, 18, 24 and 36 months, as shown in Fig. 2. In the analysis of continuous values ​​of IDAA1c, there was no difference between the two groups, except at 18 months, when group 1 had lower levels of IDAA1c than group 2 (*p* = 0.048, Table 2). When the ISPAD criteria for partial CR diagnosis were used, the intervention group also presented a higher frequency of CR. The mean duration of CR varied between the two criteria. When compared to IDAA1c, the duration of CR with ISPAD criteria was shorter (7.5 ± 9.88 vs. 14.78 ± 13.99 months, p 0.002), in group 1 (12.85 ± 11.18 vs. 22.28 ± 11.33 months, p 0.068) and in group 2 (5.71 ± 8.99 vs. 12.28 ± 14.13 months, p 0.011).

## Discussion

This study evaluated the development of partial clinical remission, glycemic control and total insulin/kg requirement between a group that received combined therapy with single-dose ASC infusion and 2,000 daily vitamin D oral supplements for 12 months and a control group with a 36-month follow-up. All patients who underwent intervention developed partial CR of variable duration and had a significantly lower insulin requirement per kilo of body weight than the control group, despite similar glycated hemoglobin levels at all times. At the end of follow-up, the intervention group needed nearly half of the total daily insulin dose (49%) needed by the control group. The therapy was safe with scarce, mild and self-limiting adverse events.

Allogenic transplantation of ASCs proved to be a safe therapy, with an easy-to-perform protocol and being potentially effective for preserving the function of pancreatic β cells and, consequently, prolonging clinical remission. In T1D [17–19] and other autoimmune diseases, such as rheumatoid arthritis [20, 21], systemic lupus erythematosus [22], inflammatory bowel disease [23], and multiple sclerosis [24], mesenchymal stem cell infusion, including ASCs, were safe, without serious adverse events with a follow-up period up to 48 weeks. In the present study, most patients presented mild and transient reactions, such as tachycardia during infusion and transient thrombophlebitis, which could be associated with high cellularity concentration, high viscosity or other cell stabilizing products. The central vein occlusion presented by one patient 3 months after infusion and the recurrence of a benign teratoma in another patient 9 months after the proposed treatment were most likely unrelated to the ASC infusion, since they occurred months after the intervention [15, 16]. Despite its potential capacity to differentiate into mesodermal and other embryonic lineages, in vitro and in vivo studies show no evidence of tumor development [25].

Several studies also sought an intervention in patients with recent-onset T1D with vitamin D supplementation. Most of these studies did not show conclusive results. However, Gabbay et al. demonstrated a significantly smaller decline in C-peptide in patients who received supplementation with 2,000 IU/day of vitamin D for 18 months, when compared to the placebo group [10]. Due to the complexity of the pathophysiology of T1D, the combination of more than one intervention is more prone to result in beneficial outcomes. In the present study, patients undergoing the combined intervention of ASC infusion with vitamin D had a higher frequency of clinical remission as well as a longer CR duration than the control group. Even though glycemic control remained similar throughout the study between the two groups, a significantly lower total insulin/kg dose needed three years after intervention suggests greater glycemic stability and a long-term benefit of intervention.

Despite the heterogeneity of criteria for defining partial CR in the literature, IDAA1c is considered the current gold standard in the definition of partial CR and was chosen to assess the presence of clinical remission in the study groups. IDAA1c values ​​with a result equal to or less than 9 have a strong correlation with the residual function of β cells estimated by levels of stimulated C-peptide above 300 pmol/L in mixed meal tests [13]. However, this threshold has been arbitrarily defined based on the range used by the Diabetes Control and Complications Trial (DCCT) for “C-peptide responders” (200–500 pmol/L) [26] and therefore may not be accurate to assess CR. Furthermore, the use of stimulated C-peptide to assess the presence of CR has been questioned, due to several limitations related to the assessment of pancreatic residual beta cell function by this method. As it is based on C-peptide values, IDAA1c also receives criticism due to its accuracy. However, since IDAA1c also evaluates the total daily dose of insulin, it provides more information about daily insulin requirements, body weight, and exercise, being more interesting than stimulated C-peptide alone [27]. Limitations of stimulated C-peptide measurement to determine CR in patients with T1D include biochemical factors, related to the variability of C-peptide stability according to assay methods [28] and its easy degradation when stored [29], which can lead to false negative results. Newly diagnosed T1D individuals have a reduced response of the GIP/GLP-1 system [30]. For correct dosing of the C-peptide stimulated through a mixed meal tolerance test, this incretin axis must be intact [31]. Therefore, the use of stimulated C-peptide in these patients may underestimate residual β cell function [32]. Finally, stimulated C-peptide alone is unable to detect changes in insulin resistance, insulin sensitivity, daily insulin requirements and patient’s body weight. Therefore, it would be important to investigate IDAA1c as a continuous variable as well to investigate other definitions of CR. The use of an insulin sensitivity score (ISS), another functional test, combined with IDAA1c, has been proposed as a measure for greater accuracy in the assessment of CR [27].

In this study, we also used the ISPAD criterion for CR [33], which indeed underestimated the presence of partial CR when compared to IDAA1c [13], with a shorter duration of partial CR in both groups and a smaller number of patients in group 2, who evolved to CR.

The percentage of patients in the control group who evolved to partial CR (47.6%) is compatible with Brazilian data in a recent study carried out by Camilo et al., which demonstrated a prevalence of 41.2% of partial CR in a cohort of 51 individuals aged between 5 and 15 years followed for 13 months after the diagnosis of T1D [34] without intervention.

This study has some limitations. First, it was a retrospective study, with a limited sample size, which may have influenced the results. The age difference at T1D onset could be considered a limitation of this study. However, most patients in each group were young adults and all patients selected to participate in the study were in a post-pubertal stage, diagnosed from the age of 16. There are differences in the rates of clinical remission and C-peptide between patients diagnosed with T1D as children, adolescents during puberty or adults. Nonetheless, these differences occur mainly between children/adolescents in the pre-pubertal/pubertal stages and those in post-pubertal stage, with patients diagnosed with T1D after puberty showing a higher incidence of spontaneous CR [35]. Moreover, a recent longitudinal cross-sectional analysis published in 2023 by Harsunen et al. [36] showed that age at diagnosis over 16 years old, among other factors, was related to a slower decline in C-peptide titers. Therefore, T1D diagnosed in a post-pubertal stage has a similar incidence of CR to that of adults [35, 36]. In addition, patients in both groups diagnosed with T1D between 16 and 18 years of age experienced clinical remission for some time. Another limitation was the lack of vitamin D assessment in most patients in the control group, whose data was retrospectively collected and after 12 months in the intervention group and controls of the previous pilot studies [12, 15]. In addition, there was no measurement of C-peptide or immunological markers during follow-up. As we did not include patients that received only one intervention (vitamin D or ASC), it was not possible to evaluate the individual role of each treatment. However, this study showed a significant reduction in the total insulin dose per kilogram needed by patients in the intervention group, with potential clinical and economic benefits, requiring further research with a larger number of participants.

The reduction in insulin requirement observed in this study might be relevant for patients with T1D. In 2022, there were 588,800 people living with T1D in Brazil [37]. Despite Brazilian’s universal health system providing human insulin, the free supply of fast-acting analogs is only performed within strict criteria, and the patient often has to bear the high costs of a quality insulin therapy, which can compromise a large part of the family budget. Longer follow-up of these patients would be important, with a larger sample size to assess the economic impact these results could have, both at the public health level and at the individual level.

Finally, interventions that increase the possibility of the individual entering into the partial CR phase are extremely relevant, since recent studies demonstrate that patients who evolved to partial CR presented several benefits, such as a lower incidence of vascular complications [38] and better insulin sensitivity, with greater glycemic stability and lower risk of severe hypoglycemia [4, 39]. Immunointerventions, such as anti-CD3 monoclonal antibodies (teplizumab) [40], transplantation of autologous Tregs [41], anti-thymocyte globulin [42] and CTLA-4-immunoglobulin (abatacept) [43], have been tested in T1D patients in pre-clinical stage 2 and recently diagnosed, in stage 3, for this purpose. Results show a slower decline of c-peptide titers, but still with limited efficacy. The treatment reported in this study may be another component to enable immunointervention in this population, and should be studied in association with other treatments and in earlier stages of the disease, such as preclinical stage 2.

## Conclusion

The present study demonstrated that the intervention with infusion of ASC + vitamin D supplementation was associated with partial CR at 6 months. Although there were no differences in CR established by the IDAA1c and ISPAD criteria after three years of follow-up, patients who underwent intervention had 49% lower insulin requirement than controls with conventional treatment, with similar glycemic control. Further studies with a larger sample and longer follow-up are still needed to better assess these outcomes.

## Data Availability

The datasets used and/or analysed during the current study are available from the corresponding author on reasonable request.

## Abbreviations

T1D: type 1 diabetes
CR: clinical remission
ASC: adipose-tissue derived mesenchymal stem cell
UFRJ: Federal University of Rio de Janeiro
DKA: diabetic ketoacidosis
IDAA1c: insulin-dose adjusted A1c
ISPAD: International Society of Pediatric and Adolescent Diabetes
HbA1c: glycated hemoglobin
UI: international units
Kg: kilograms
HPLC: High Performance Liquid Chromatography by boronate affinity
HUCFF: Clementino Fraga Filho University Hospital
DCCT: Diabetes Control and Complications Trial
ISS: insulin sensitive score
